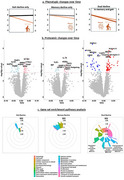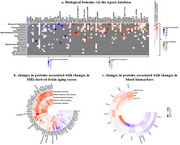# Proteomic Signatures of Dual Cognitive and Mobility Decline in Older Adults

**DOI:** 10.1002/alz70856_100402

**Published:** 2025-12-24

**Authors:** Qu Tian, Erin E Greig, Michael R. Duggan, Keenan A. Walker, Luigi Ferrucci

**Affiliations:** ^1^ National Institute on Aging, Baltimore, MD, USA; ^2^ Laboratory of Behavioral Neuroscience, National Institute on Aging, Intramural Research Program, Baltimore, MD, USA; ^3^ Translational Gerontology Branch, National Institute on Aging, NIH, Baltimore, MD, USA

## Abstract

**Background:**

Across diverse aging populations worldwide, dual memory and gait decline is consistently associated with substantially higher dementia risk than memory decline only. Understanding underlying mechanisms is key to the early detection of at‐risk populations and developing prevention strategies. Identifying concurrent and longitudinal proteomic signatures of dual decline provides novel insights into underlying biological processes.

**Method:**

We compared 7,268 plasma proteomic makers via the SomaScan platform cross‐sectionally and longitudinally between dual decline (*n* = 82), memory decline (*n* = 93), gait decline (*n* = 100), and no decline (*n* = 251)(Figure 1a) in Baltimore Longitudinal Study of Aging participants aged 60 and older using linear mixed‐effects regression, adjusted for demographics. Based on proteins related to each declining group, we identified biological domains via the Agora database. We further performed gene set enrichment pathway analyses (GSEA). We also examined associations of proteomic signatures with MRI‐derived brain aging scores (subcortical, medial temporal lobe, parieto‐temporal, diffuse cortical, perisylvian) and blood biomarkers of AD and neurodegeneration (Aβ_42/40_, pTau181, GFAP, NfL) using multivariable linear regression.

**Result:**

No cross‐sectional proteomic differences were found between any declining group and the no decline group. Longitudinally, compared to no decline, only the dual decline group showed significant changes in 75 proteins (FDR‐p<0.05), notably in biological domains of structural stabilization, synapse, immune response, proteostasis, and lipid metabolism (FDR‐p<0.05) (Figure 1b, 2a). GSEA revealed the top enriched pathway of mitochondrial protein degradation (FDR‐p<0.0001) (Figure 1b). Longitudinal changes of selected proteins were associated with accelerated brain atrophy longitudinally in the medial temporal lobe (38 proteins), subcortical (30 proteins), perisylvian (29 proteins), diffuse cortical (26 proteins), and parieto‐temporal areas (7 proteins) (all *p* <0.05). Longitudinal changes in 12 proteins were associated with changes in biomarkers of AD and neurodegeneration, including TRI72 (β=3.751, *p* = 0.008) and TREM2 (β=‐3.000, *p* = 0.022) which showed the strongest associations with the progression of pTau181 (Figure 2b).

**Conclusion:**

Older adults experiencing dual decline exhibit characteristic changes in plasma proteins over time. The biological processes connecting dual decline with high dementia risk may be related to mitochondrial function, synaptic function, proteostasis, and immune responses. Future studies are warranted to confirm these findings in other aging cohorts.